# Patterns of Lymph Node Metastasis in Patients With T1/T2 Gastroduodenal Neuroendocrine Neoplasms: Implications for Endoscopic Treatment

**DOI:** 10.3389/fendo.2021.658392

**Published:** 2021-05-28

**Authors:** Yu-Jie Zhou, Qi-Wen Wang, Qing-Wei Zhang, Jin-Nan Chen, Xin-Yuan Wang, Yun-Jie Gao, Xiao-Bo Li

**Affiliations:** Division of Gastroenterology and Hepatology, Key Laboratory of Gastroenterology and Hepatology, Ministry of Health, Shanghai Institute of Digestive Disease, Renji Hospital, School of Medicine, Shanghai Jiao Tong University, Shanghai, China

**Keywords:** upper gastrointestinal tract, neuroendocrine neoplasm, lymph node metastasis, endoscopic resection, duodenum

## Abstract

Guidelines have differed in their opinion regarding the indications for endoscopic resection of gastric-neuroendocrine neoplasms (g-NENs) and duodenal-NENs (d-NENs). We examined the association between size and lymph node metastasis (LNM) to identify candidates most suitable for endoscopic resection. We identified 706 patients with T1/T2 g-NENs and 621 patients with T1/T2 d-NENs from the SEER database. The prevalence of LNM and risk factors associated with LNM were analyzed. LNM was present in 8.1% of patients with gastroduodenal neuroendocrine tumors (NETs) and 31.6% of patients with neuroendocrine carcinomas (NECs). Multivariate logistic regression indicated that tumor size >10mm, greater invasion depth, and poor differentiation were independently associated with LNM. In addition, the percentage of g-NETs invading submucosa with LNM increased with tumor size (≤10 mm,3.9%;11–20 mm,8.6%;>20 mm,16.1%). However, in contrast to the low LNM risk in patients with small g-NETs (≤10 mm), we found that LNM rate exceeded 5% even for patients with small submucosal-infiltrating d-NETs. Among patients with nodal-negative g-NETs, the cause specific survival (CSS) was similar for those who received surgical resection and endoscopic resection. Among patients with d-NETs, the CSS was better for those who received endoscopic resection. In conclusion, patients with d-NETs had a higher probability of LNM than those with g-NETs. Endoscopic resection can be utilized for curative treatment of submucosa-infiltrating g-NETs and intramucosal d-NETs when the size is 10 mm or less. These results reinforce the need to search for LNM in lesions that are larger than 10 mm.

## Introduction

Gastroduodenal neuroendocrine neoplasms (NENs), including gastric neuroendocrine neoplasms (g-NENs) and duodenal neuroendocrine neoplasms (d-NENs), account for approximately 10% of NENs within the digestive system (1, 2). With advances in endoscopic techniques, clinicians now incidentally detect an increasing number of gastroduodenal NENs and remove them endoscopically at an early stage (3, 4). Similar to early gastric cancer, the potential risk of lymph node metastasis (LNM) must be considered before endoscopic resection. There are some differences in the guidelines of major European and North American societies regarding the endoscopic management of superficial gastroduodenal NENs (5–7).

Most g-NENs arise from enterochromaffin-like (ECL) cells and multiple guidelines classify them into three types: type 1 (70–80%) is associated with autoimmune gastritis, type 2 (5–6%) results from gastrinoma, and type 3 (14–25%) occurs without hypergastrinaemia (8). The current European Neuroendocrine Tumor Society (ENETS) consensus guidelines consider patients with type 1 g-NENs larger than 10 mm to have an increased risk of metastasis. However, the guidelines of the North American Neuroendocrine Tumor Society (NANETS) recommend that type 1 and 2 g-NENs that are confined to the submucosa, less than 20 mm in diameter, and with no more than six polyps could be resected endoscopically (5). Moreover, the most recent National Comprehensive Cancer Network (NCCN) guidelines suggested that endoscopic resection should be reserved for small (<10 mm), low-grade, and superficial g-NENs (9). Thus, there are differences in whether endoscopic resection should be utilized for curative treatment of g-NENs with diameters of 10 to 20 mm.

More than 90% of d-NENs are in the first or second part of the duodenum, and they are generally small (1.2–1.5 cm) and solitary (8, 10). The ENETS guidelines state that nonampullary d-NENs less than 10 mm in diameter and confined to the submucosal layer are candidates for endoscopic treatment. However, there is no consensus regarding the use of endoscopic or surgical resection for d-NENs that are 10 to 20 mm in diameter (6), and the NANETS and NCCN guidelines do not specifically refer to endoscopic management of d-NENs.

In this study, we examined the indications for endoscopic resection of T1/T2 g-NENs and d-NENs, with a focus on neoplasms that are 10 to 20 mm in diameter, by determining the relationship between the size of gastroduodenal NENs and the prevalence of LNM in a large population.

## Materials and Methods

### Study Population

We retrieved clinicopathological data of all patients who were diagnosed with a g-NEN or d-NEN between 2004 and 2015 from National Cancer Institute-sponsored Surveillance, Epidemiology, and End Results (SEER) Program (www.seer.cancer.gov). This registry has research data from 1975 to 2016, and was released in April 2019. The annually-updated SEER database is one of the largest registries in the world, consists of 18 population-based cancer registries, and comprises about 28% of all US cancer cases (11). According to American Joint Committee on Cancer (AJCC) 8^th^ TNM staging system for g-NEN and d-NEN, a T1 tumor is one that has invaded the lamina propria or submucosa and is 10 mm or less in size, and a T2 tumor is one that has invaded the muscularis propria or is more than 10 mm in size (12).

The inclusion criteria were: NEN as the primary tumor; histologically confirmed gastroduodenal NEN with International Classification of Diseases (ICD) histologic codes of 8240 to 8249, 8152 to 8156, 8013, or 8041; primary site ICD code of C16.0 to C16.9 for g-NEN, and C17.0 for d-NEN; and stage T1 or T2. The exclusion criteria were: missing information on tumor size, tumor grade, or lymph node metastasis status; unknown T stage or stage T3 or T4; presence of distant metastasis; receipt of preoperative radiotherapy; and a d-NEN located in the ampulla of Vater.

### Definitions of NET and NEC

The term ‘NEN’ refers to two groups of neoplasms with distinct prognoses: neuroendocrine tumor (NET) and neuroendocrine carcinoma (NEC). This study followed the 2019 WHO classification and grading criteria for tumors of the gastrointestinal tract (13), which defined well or moderately differentiated gastric and duodenal NENs as ‘g-NETs’ and ‘d-NETs’, and poorly differentiated gastroduodenal NENs as ‘g-NECs’ and ‘d-NECs’. We referred to the LNM rate of T1a early gastric cancer (14), which is deed as an entity with low risk of LNM, to define the ‘low LNM risk’ as <5% for gastroduodenal NETs in this study.

### Survival Analysis

Surgery of SEER primary site code 20 to 27 was defined as local tumor excision (endoscopic treatment including polypectomy, excisional biopsy, and electrocautery) and code 30 to 90 as open surgery. Nodal involvement was determined by examination of lymph nodes during open surgery or by pre-resection imaging (endoscopic ultrasound, computed tomography scanning, or magnetic resonance imaging) for patients who received endoscopic treatment (15). Cause-specific survival (CSS) was defined as the time from diagnosis to death from gastroduodenal NEN. Multivariate Cox regression analysis for two end points — overall survival (OS) and CSS — was used to assess the prognostic effects of age, sex, tumor size, and treatment method for patients with nodal-negative NETs that were confined to the submucosa. Survival curves were plotted using the Kaplan-Meier method, and the log-rank test was utilized to determine significance of differences for two comparisons: nodal-negative patients who underwent endoscopic excision *vs.* open surgery and patients with LNM *vs.* no LNM.

### Statistical Analysis

Continuous variables were presented as means ± standard deviations, and categorical data as numbers and percentages. Continuous variables with or without normal distribution were compared using Student’s *t*-test or the two-sample Mann-Whitney U test, as appropriate. Categorical data were compared using the chi-squared test or Fisher’s exact test, as appropriate. Stratified categorical data were compared using the Cochran-Mantel-Haenszel test. A multivariate logistic regression model was employed to identify factors independently associated with LNM. All statistical analyses were performed by IBM SPSS version 22.0 and R version 3.6.1 (https://www.r-project.org/). For all statistical tests, a two-sided *P* value less than 0.05 was regarded significant.

## Results

### Clinical Characteristics of Patients

We retrospectively examined 1327 patients from the SEER database who had stage T1/T2 gastroduodenal NENs and were diagnosed between 2004 and 2015 (**Tables 1** and **2**). There were 706 patients with g-NENs and 621 patients with d-NENs, and the average age at diagnosis was 59.5 ± 12.9 years for those with g-NENs and 62.2 ± 12.0 years for those with d-NENs. Females accounted for 62.6% of g-NEN patients and 50.5% of d-NEN patients. A total of 95.5% of g-NENs were g-NETs with good differentiation, and 99% of d-NENs were d-NETs. The overall prevalence of LNM was 5.8% among those with g-NENs and 12.1% among those with d-NENs. Analysis of multiple clinicopathological variables indicated LNM had significant associations with male sex, early diagnosis, poor differentiation, deep invasion, and tumor size in patients with g-NENs and d-NENs (all *P* < 0.05). Our comparison of patients with and without LNM also indicated differences in race/ethnicity of those with g-NENs (*P* = 0.03) and age differences in those with d-NEN (*P* < 0.001). However, g-NEN location was not associated with LNM (*P* = 0.21).

**Table 1:** Characteristics of patients with T1/T2 g-NENs (n = 706) with and without LNM.

Variable	No LNM (n = 665)	With LNM (n = 41)	*P*
Age at diagnosis (mean ± SD)	59.3 ± 13.0	62.2 ± 9.8	0.17
Male (%)	238 (35.8%)	26 (63.4%)	<0.001
Year of diagnosis			<0.001
2004-2007	26 (3.9%)	8 (19.5%)	
2008-2011	151 (22.7%)	11 (26.8%)	
2012-2015	488 (73.4%)	22 (53.7%)	
Race/Ethnicity			0.03
Non-Hispanic White	381 (57.3%)	22 (53.7%)	
Black	95 (14.3%)	5 (12.2%)	
Hispanic White	43 (21.5%)	6 (14.6%)	
Asian/Pacific Islanders	32 (4.8%)	7 (17.1%)	
American Indian/Alaska Native	5 (0.8%)	0	
Unknown	9 (1.4%)	1 (2.4%)	
Tumor differentiation			<0.001
Well differentiated	531 (79.8%)	24 (58.5%)	
Moderately differentiated	112 (16.8%)	7 (17.1%)	
Poorly differentiated (NEC)	22 (3.3%)	10 (24.4%)	
Depth of invasion			<0.001
Mucosa	153 (23.0%)	3 (7.3%)	
Submucosa	267 (40.2%)	20 (48.8%)	
Muscularis propria	84 (12.6%)	14 (34.1%)	
T1, NOS	129 (19.4%)	0	
T2, NOS	32 (4.8%)	4 (9.8%)	
Tumor size			<0.001
≤10 mm	452 (68.0%)	9 (22.0%)	
11- 20 mm	136 (20.5%)	10 (24.4%)	
21- 50 mm	68 (10.2%)	14 (34.1%)	
>50 mm	9 (1.3%)	8 (19.5%)	
Location			0.21
Cardia/Fundus	105 (15.8%)	8 (19.5%)	
Body	219 (32.9%)	10 (24.4%)	
Antrum/Pylorus	145 (21.8%)	14 (34.1%)	
Stomach, NOS	196 (29.5%)	9 (22.0%)	

LNM, lymph node metastasis; g-NEN, gastric neuroendocrine neoplasm; SD, standard deviation; NEC, neuroendocrine carcinoma; NOS, Not otherwise specified.

**Table 2:** Characteristics of patients with T1/T2 d-NENs (n = 621) with and without LNM.

Variable	No LNM (n = 546)	With LNM (n = 75)	*P*
Age at diagnosis (mean ± SD)	63.0 ± 11.9	56.8 ± 11.9	<0.001
Male (%)	280 (51.3%)	29 (38.7%)	0.04
Year of diagnosis			<0.001
2004-2007	19 (3.5%)	6 (8.0%)	
2008-2011	110 (20.1%)	28 (37.3%)	
2012-2015	417 (76.4%)	41 (54.7%)	
Race/Ethnicity			0.39
Non-Hispanic White	290 (53.1%)	49 (65.3%)	
Black	150 (27.5%)	16 (21.3%)	
Hispanic White	61 (11.2%)	4 (5.3%)	
Asian/Pacific Islanders	37 (6.8%)	5 (6.7%)	
American Indian/Alaska Native	2 (0.4%)	0	
Unknown	6 (1.1%)	1 (1.3%)	
Tumor differentiation			0.02
Well differentiated	478 (87.5%)	57 (76.0%)	
Moderately differentiated	64 (11.7%)	16 (21.3%)	
Poorly differentiated (NEC)	4 (0.7%)	2 (2.7%)	
Depth of invasion			<0.001
Mucosa	191 (35.0%)	10 (13.3%)	
Submucosa	280 (51.3%)	26 (34.7%)	
Muscularis propria	51 (9.3%)	36 (48.0%)	
T1, NOS	18 (3.3%)	1 (1.3%)	
T2, NOS	6 (1.1%)	2 (2.7%)	
Tumor size			<0.001
≤10 mm	404 (74.0%)	29 (37.3%)	
11- 20 mm	117 (21.4%)	30 (40.0%)	
21- 50 mm	17 (3.1%)	15 (20.0%)	
>50 mm	8 (1.5%)	2 (2.7%)	

LNM, lymph node metastasis; d-NEN, duodenal neuroendocrine neoplasm; SD, standard deviation; NEC, neuroendocrine carcinoma; NOS, Not otherwise specified.

### Risk Factors for Lymph Node Involvement

We also examined potential risk factors for LNM in patients with gastroduodenal NENs (**Tables 3** and **4**). To avoid the effect of multicollinearity among tumor size, invasion depth, and tumor differentiation (*P* < 0.001 identified by Spearman’s correlation analyses, data not shown), we first employed univariate logistic regression and then used a multivariate model that adjusted for demographic factors (age, sex, and race). The results indicated that tumor size greater than 10 mm, depth of invasion, and poor differentiation were risk factors for LNM in patients with g-NENs in the unadjusted and multivariable logistic regression (all *P* < 0.05). The results were similar for patients with d-NENs.

**Table 3:** Logistic regression of factors associated with LNM in patients with g-NENs.

Variable	Model 1	*P*	Model 2	*P*
	OR (95% CI)		OR (95% CI)	
Tumor differentiation				
Well differentiated	Reference	–	Reference	–
Moderately differentiated	1.38 (0.58-3.29)	0.46	1.37 (0.57-3.27)	0.48
Poorly differentiated (NEC)	10.06 (4.29-23.58)	<0.001	8.38 (3.41-20.58)	<0.001
Depth of invasion*				
Mucosa	Reference	–	Reference	–
Submucosa	3.82 (1.12-13.07)	0.03	3.92 (1.14-13.54)	0.03
Muscularis propria	8.50 (2.38-30.42)	0.001	8.82 (2.44-31.96)	0.001
Tumor size				
≤10 mm	Reference	–	Reference	–
11- 20 mm	3.69 (1.47-9.27)	0.005	3.96 (1.54-10.20)	0.004
>20 mm	14.35 (6.37-32.33)	<0.001	17.38 (7.38-40.92)	<0.001

*Cohort size, n = 541 (data on specific layer of invasion depth were available only in 541 out of 706 g-NEN patients). Logistic regression was utilized to explore the association of tumor differentiation, size, and invasive depth with LNM in model 1 (univariate). Confounding variables of age, sex, and race were adjusted in model 2. LNM, lymph node metastasis; g-NEN, gastric neuroendocrine neoplasm; OR, odds ratio; CI, confidence interval.

**Table 4:** Logistic regression of factors associated with LNM in patients with d-NENs.

Variable	Model 1	*P*	Model 2	*P*
	OR (95% CI)		OR (95% CI)	
Tumor differentiation				
Well differentiated	Reference	–	Reference	–
Moderately differentiated	2.10 (1.14-3.87)	0.02	1.94 (1.02-3.67)	0.04
Poorly differentiated (NEC)	4.19 (0.75-23.40)	0.10	5.71 (0.95-34.19)	0.06
Depth of invasion*				
Mucosa	Reference	–	Reference	–
Submucosa	1.77 (0.84-3.76)	0.14	1.86 (0.87-4.00)	0.11
Muscularis propria	13.48 (6.27-28.99)	<0.001	13.18 (6.00-28.95)	<0.001
Tumor size				
≤10 mm	Reference	–	Reference	–
11- 20 mm	3.70 (2.13-6.44)	<0.001	4.11 (2.31-7.33)	<0.001
>20 mm	9.81 (4.75-20.27)	<0.001	9.73 (4.52-20.95)	<0.001

*Cohort size, n = 594 (data on specific layer of invasion depth were available only in 594 out of 621 d-NEN patients). Logistic regression was utilized to explore the association of tumor differentiation, size, and invasive depth with LNM in model 1 (univariate). Confounding variables of age, sex, and race were adjusted in model 2. LNM, lymph node metastasis; d-NEN, duodenal neuroendocrine neoplasm; OR, odds ratio; CI, confidence interval.

Our results also indicated that tumor size (stratified as ≤10, 10–20, and >20 mm) had a high correlation with LNM in patients with g-NENs and d-NENs (both *P* < 0.001 for trend). Notably, compared with NENs of 10 mm or less in diameter, the risk of LNM was significantly increased in patients with tumors that were 11 to 20 mm in diameter among those with g-NENs [adjusted odds ratio (aOR): 3.96, 95% confidence interval (CI): 1.54–10.2; *P* = 0.004] and among those with d-NENs (aOR: 4.11, 95% CI: 2.31–7.33; *P* < 0.001).

### Prevalence of LNM in NETs With Different Sizes

**Figure 1:**
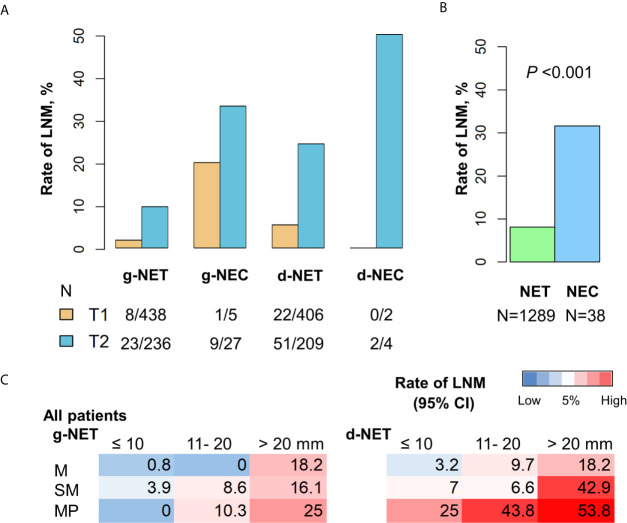
Incidence of LNM in patients with g-NENs and d-NENs. **(A)** LNM rates in T1 and T2 stages stratified by tumor differentiation. **(B)** Comparison of LNM rates in all NETs (n = 1289) and all NECs (n = 38). **(C)** Risk of LNM in NETs with different invasion depths and sizes. g-NEN, gastric neuroendocrine neoplasm; d-NEN, duodenal neuroendocrine neoplasm; LNM, lymph node metastasis; NET, neuroendocrine tumor; NEC, neuroendocrine carcinoma.

Overall, 104 of 1289 patients with gastroduodenal NETs (8.1%) and 12 of 38 patients with NECs (31.6%) had LNM. Thus, as expected, the prevalence of LNM was greater in patients with NEC (Chi-square *P* < 0.001, **Figures 1A, B**). Analysis of NECs indicated there was no LNM in the 2 patients with T1 d-NEC, but LNM was present in 20.0% of patients with T1 g-NEC, 33.3% of patients with T2 g-NEC, and 50.0% of patients with T2 d-NEC. Analysis of patients with NETs indicated that LNM was present in 1.8% of patients with T1 g-NET, 9.7% of patients with T2 g-NET, 5.4% of patients with T1 d-NET, and 24.4% of patients with T2 d-NET. Thus, patients with T1/T2 gastroduodenal NECs are not optimal candidates for endoscopic resection due to the high prevalence of LNM. We therefore focused on T1/T2 gastroduodenal NETs in our subsequent analysis.

We examined the association of invasion depth, tumor size, and LNM in patients with gastroduodenal NETs for whom data on invasion depth were available (**Table 5**). Among 517 patients with g-NETs, LNM status was detected in 254 patients using cross-sectional imaging or endoscopic ultrasound (EUS) and in 263 patients by open surgery. Analysis of the 517 patients with g-NETs indicated LNM occurred in 1.9% of patients with tumors in the mucosa, 6.4% of patients with tumors in the submucosa, and 12.3% of patients with tumors in the muscularis propria. Among 589 patients with d-NETs, LNM status was detected in 288 patients using cross-sectional imaging or EUS and in 301 patients by open surgery. Analysis of the 589 patients with d-NETs indicated LMN occurred in 5% of patients with tumors in the mucosa, 8.6% of patients with tumors in the submucosa, and 40.0% of patients with tumors in the muscularis propria. Our analysis of the relationship of LNM in each layer with tumor size indicated a high risk for LNM (nodal metastasis rate > 5%) in 8 of the 9 subgroups of patients with d-NETs, but not in the subgroup with mucosal tumors smaller than 10 mm. There was also a high risk for LNM in 5 of the 9 subgroups of patients with g-NETs, but not in the 3 subgroups with tumors smaller than 10 mm or in the subgroup with mucosal tumors that were 11 to 20 mm (**Table 5** and **Figure 1C**).

**Table 5:** Association of invasion depth, tumor size, and LNM in 1106 patients with gastroduodenal NETs.

Invasion depth	Prevalence of LNM				*P*
	Total	≤10 mm	11-20 mm	>20 mm	
g-NETs (n = 517)					<0.001
Mucosa	3/155 (1.9%)	1/130 (0.8%)	0/14	2/11 (18.2%)	
Submucosa	18/281 (6.4%)	7/180 (3.9%)	6/70 (8.6%)	5/31 (16.1%)	
Muscularis propria	10/81 (12.3%)	0/18	4/39 (10.3%)	6/24 (25.0%)	
					
d-NETs (n = 589)					<0.001
Mucosa	10/200 (5.0%)	5/158 (3.2%)	3/31 (9.7%)	2/11 (18.2%)	
Submucosa	26/304 (8.6%)	16/229 (7.0%)	4/61 (6.6%)	6/14 (42.9%)	
Muscularis propria	34/85 (40.0%)	6/24 (25.0%)	21/48 (43.8%)	7/13 (53.8%)	

LNM, lymph node metastases; NET, neuroendocrine tumor; g-NEN, gastric neuroendocrine neoplasm; d-NEN, duodenal neuroendocrine neoplasm.

### NEN-Specific Survival According to Tumor Stage/Grade and Treatment Modality

**Figure 2:**
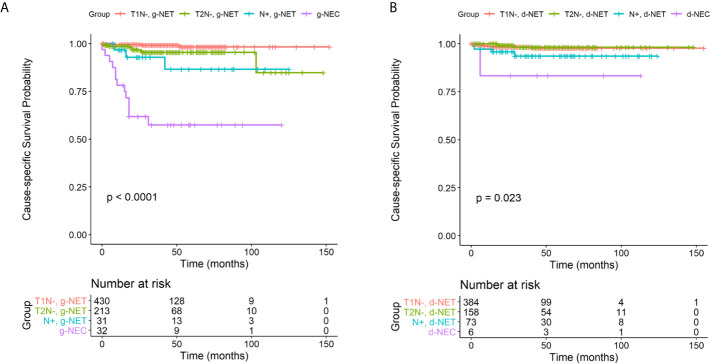
Cause-specific survival in four clinical subgroups (T1 nodal-negative NETs, T2 nodal-negative NETs, nodal-positive NETs, and NECs) among patients with g-NENs **(A)** and d-NENs **(B)**. NET, neuroendocrine tumor; NEC, neuroendocrine carcinoma; g-NEN, gastric neuroendocrine neoplasm; d-NEN, duodenal neuroendocrine neoplasm.

Patients with g-NENs had a median follow-up period of 43.7 months and an overall 5-year survival rate of 84.0%. Patients with d-NENs had a median follow-up period of 43.2 months and an overall 5-year survival of 87.1%. We then determined CSS analysis on four different subgroups for patients with g-NENs and d-NENs: T1 nodal-negative NETs, T2 nodal-negative, nodal-positive NETs, and NECs (**Figure 2**). The results indicated NEN-specific survival for NEC patients was significantly worse than the other 3 subgroups among patients with d-NENs (*P* < 0.001) and among patients with g-NENs (*P* = 0.02). However, the CSS was not significantly different in nodal-negative NET patients with stage T1 or T2. Univariate and multivariate Cox regression analysis indicated that LNM increased the risk for poor OS and CSS in patients with T1/T2 g-NETs (adjusted hazard ratio (aHR): 6.15, 95% CI: 2.67–14.1; *P* < 0.001; **Supplementary Table 1**). In addition, analysis of patients with T1/T2 d-NETs indicated that increased age was associated with worse OS (aHR: 1.05, 95% CI: 1.03–1.08; *P* < 0.001) and nodal involvement was associated with decreased CSS (aHR: 4.25, 95% CI: 1.24–14.5; *P* = 0.02).

Next, we compared the CSS rates of patients who underwent local excision (endoscopic treatment including polypectomy, excisional biopsy, and electrocautery) or surgical resection. There were 470 patients with nodal-negative g-NETs and 400 patients with nodal-negative d-NETs with tumors confined to submucosal layer for whom information on treatment was available. Among them, 587 patients (67.5%) received local excision and 283 patients (32.5%) received radical surgery. For patients with g-NETs, the unadjusted Kaplan-Meier curves were similar in the two groups (**Supplementary Figure 1A**). However, for patients with d-NETs, those who received surgical resection had a significantly worse CSS (*P* = 0.004; **Supplementary Figure 1B**). In agreement, Cox regression analysis with adjustment for age, sex, and tumor size also demonstrated that open surgical treatment was associated with poor CSS in patients with nodal-negative d-NETs confined to the submucosa (**Supplementary Table 2**).

## Discussion

This large population-based study examined risk factors for LNM in patients who had T1/T2 upper gastrointestinal NENs. We found that tumor differentiation, size, and infiltration depth were significantly associated with LNM. Patients with poorly-differentiated NECs had a high risk of LNM, indicating that endoscopic resection was an inappropriate treatment. Thus we further investigated the association of tumor size, tumor invasion, and LNM in patients with early gastroduodenal NETs to identify the suitability of endoscopic resection.

Our findings indicated that patients with a tumor of 11 to 20 mm had a higher risk of LNM than patients with a tumor than 10 mm for those with early-stage g-NETs or early-stage d-NETs. As such, it should be prudent to perform endoscopic resection of intermediate-sized gastroduodenal NETs (11–20 mm); EUS and abdominal enhancement computed tomography are needed to assess tumor infiltration and LNM for patients with these NETs. Our results suggest that surgical resection is most appropriate when the tumor is larger than 20 mm. In contrast to early gastrointestinal cancer, NETs originate from endocrine cells in the deep mucosa. Previous research showed that cold biopsy forceps polypectomy was inadequate for curative treatment of gastrointestinal NETs because of the high rates of submucosal infiltration. Instead, pathological examination after complete resection by snare polypectomy with electrocauterization, endoscopic mucosal resection (EMR), or endoscopic submucosal dissection (ESD) are appropriate alternatives for biopsy of NETs (16). ESD is a safe and effective procedure that provides accurate pathological assessment and curative treatment for patients with upper gastrointestinal NETs (4, 17, 18). In our opinion, patients with intermediate-sized NETs who are willing to receive endoscopic resection should be informed of a risk for the need of additional open surgery, and diagnostic EMR/ESD can be performed after careful evaluation. As pointed out in ENETS guidelines, EUS should be performed for g-NETs larger than 10 mm before endoscopic excision. Even if curative endoscopic resection is achieved, regular follow-ups are important for patients whose gastroduodenal NETs were larger than 10 mm. If the pathological examination of ESD specimen shows lymphvascular invasion or muscularis propria invasion, additional surgical resection with lymph node dissection is necessary for NET patients, considering worse prognosis of recurrence in regional lymph node (19).

There are five clinical entities of d-NENs, and the two main ones are gastrinomas (non-functional neoplasms with positive neuroendocrine markers) and somatostatinomas. Duodenal gastrinomas are associated with multiple endocrine neoplasia type 1, and somatostatinomas often occur in the periampullary region (10). The term ‘d-NEN’ in the present study excludes tumors in the ampulla of Vater, because these tumors cannot be resected using endoscopy. A previous study reported that patients with d-NENs had better prognosis but a higher probability of regional LNM than those with g-NENs (20). We also observed higher rates of LNM of d-NETs than NETs from the stomach (5.4% *vs.* 1.8% for T1, 24.4% *vs.* 9.7% for T2). In addition, consistent with previous reports that less than 1 to 3% of d-NENs are poorly differentiated (21), nearly all d-NENs in our study (615 of 621) were well-differentiated. This might be related to the more favorable outcomes of these patients. To date, there is no consensus about the association between tumor size and the prevalence of LNM in patients with NETs in the duodenum due to the rarity of this clinical entity (21). As expected, we found that the incidence of LNM increased as tumor size increased. However, in contrast to the low risk of LNM in patients with small g-NETs, we found that the rate of LNM exceeded 5% even for patients with small d-NETs (≤10 mm) that were in the submucosal layer. In other words, the risk of LNM in patients with d-NETs that are less than 10 mm in diameter must be considered when selecting a treatment.

We also compared the long-term survival of patients with upper gastrointestinal NETs who underwent endoscopic resection *vs.* surgical resection. Our results showed that patients in these two groups had similar survival when they had nodal-negative g-NETs that were confined to the submucosa, but there was improved CSS for patients with d-NETs who underwent endoscopic treatment. A possible explanation for this phenomenon is that some surgical complications resulted from large-scale surgical resection caused worse prognosis in some elderly patients. However, the risk of perforation is higher for endoscopic resection of d-NETs because the bowel wall of the duodenum is thinner (22). A previous study reported a perforation rate of about 6 to 7% (18, 23). As such, although endoscopic resection of d-NETs may improve CSS by reducing the complications resulting from open surgery, considering risk of LNM and perforation, only patients with d-NETs that are small and superficial are candidates for endoscopic treatment by experienced endoscopists (24).

Some limitations should be noted in this SEER-based analysis. The major drawback was missing data on mitotic counts and Ki-67 labeling, which are widely used in clinical practice for assessment of proliferation. According to the newest WHO classification, NETs and NECs are distinguished by tumor cell differentiation, and poorly-differentiated NECs are not formally graded but are considered high-grade by definition (13). Even so, our inability to grade NETs influenced the accuracy and reliability of our findings regarding the patterns of LNM.

Another shortcoming was that we could not classify patients with g-NENs according to clinical subgroups (types 1–3 according to etiology, in which type 3 lesions have the poorest differentiation and are associated with the poorest clinical outcome). As indicated in ENETS guidelines, open surgery was recommended for type 1 g-NENs with poor differentiation, metastasis, or muscularis propria invasion, and for all type 2 and type 3 g-NENs (6, 7). Because type 1 and type 2 gastric NENs account for the vast majority of g-NETs, and because most type 3 g-NENs are g-NECs (6, 25), we tried to compensate for this limitation by separately assessing LNM in patients with NETs and NECs. Although there were still some type 3 tumors in patients within g-NETs, previous studies found that endoscopic resection of small and well-differentiated type 3 g-NETs can be curative, similar to type 1 and 2 g-NETs (26–28). Besides, the SEER database does not document the exact locations of d-NENs, and we could not exclude the possibility that some d-NENs were in the third and fourth part of the duodenum. Although neoplasms at these sites account for less than 10% of all d-NENs, these regions are inaccessible by upper endoscopy (8). In addition, information of Somatostatin Receptor Scintigraphy was lacking, which might underestimate the presence of small lymph nodal metastases. Also, the SEER database also has no data on lymph vascular involvement (LVI), which is closely associated with LNM. Because LVI is crucial for clinical judgement of curative resection after ESD (29), further studies should examine the relationship between size and grade of NETs and LVI.

In summary, our results showed that LNM was more common in patients with superficial gastroduodenal NETs in which the tumor was more than 10 mm in diameter. In light of the low LNM rate, our results support the use of endoscopic resection for curative treatment of g-NETs that are 10 mm and smaller and confined to the submucosa, as well as intramucosal d-NETs. LNM was more common in patients with d-NETs than g-NETs, and we therefore suggest that the risk of nodal involvement should considered even for submucosa-infiltrating d-NETs that are smaller than 10 mm. Further validation of these findings in a multicenter prospective study is warranted.

## Data Availability Statement

The original contributions presented in the study are included in the article/**Supplementary Material**. Further inquiries can be directed to the corresponding author.

## Ethics Statement

Written informed consent was obtained from the individual(s) for the publication of any potentially identifiable images or data included in this article.

## Author Contributions

Y-JZ and X-BL contributed to study design. Q-WZ, Q-WW, J-NC, Y-JG, and X-YW collected the study data. Y-JZ and Q-WZ contributed to data analysis and interpretation. Y-JZ contributed to manuscript writing. X-BL reviewed the manuscript and contributed to quality control. All authors contributed to the article and approved the submitted version.

## Funding

This work was supported by grants from the Program for Promoting Advanced Appropriate Technology of Shanghai Health Commission (2019SY003), National Science and Technology Major Projects of China (2018ZX10302206-004-002).

## Conflict of Interest

The authors declare that the research was conducted in the absence of any commercial or financial relationships that could be construed as a potential conflict of interest.

## Supplementary Material

Supplementary Figure 1 Cause-specific survival of nodal-negative g-NETs **(A)** and d-NETs **(B)** in the submucosal layer in groups that received endoscopic resection or open surgical resection. g-NET, gastric neuroendocrine tumor; d-NET, duodenal neuroendocrine tumor.

Supplementary Table 1A Cox proportional hazards model assessing factors associated with OS and CSS in T1/T2 g-NETs.

Supplementary Table 1B Cox proportional hazards model assessing factors associated with OS and CSS in T1/T2 d-NETs.

Supplementary Table 2A Cox proportional hazards model assessing factors associated with OS and CSS in nodal-negative g-NETs confined to the submucosa.

Supplementary Table 2B Cox proportional hazards model assessing factors associated with OS and CSS in nodal-negative d-NETs confined to the submucosa.

## Abbreviations

NEN, neuroendocrine neoplasm; g-NEN, gastric neuroendocrine neoplasm; d-NEN, duodenal neuroendocrine neoplasm; LNM, lymph node metastasis; ECL, enterochromaffin-like; ENETS, European Neuroendocrine Tumor Society; EUS, endoscopic ultrasound; NANETS, North American Neuroendocrine Tumor Society; NCCN, National Comprehensive Cancer Network; SEER, Surveillance, Epidemiology, and End Results; AJCC, American Joint Committee on Cancer; ICD, International Classification of Diseases; NET, neuroendocrine tumor; NEC, neuroendocrine carcinoma; CSS, cause-specific survival; OS, overall survival; aOR, adjusted odds ratio; CI, confidence interval; aHR, adjusted hazard ratio; EMR, endoscopic mucosal resection; ESD, endoscopic submucosal dissection; LVI, lymph vascular involvement.

## References

[1] Frilling A Akerstrom G Falconi M Pavel M Ramos J Kidd M. Neuroendocrine Tumor Disease: An Evolving Landscape. Endocr Relat Cancer (2012) 19(5):R163–85. 10.1530/ERC-12-0024 22645227

[2] Cives M Strosberg JR. Gastroenteropancreatic Neuroendocrine Tumors. CA Cancer J Clin (2018) 68(6):471–87. 10.3322/caac.21493 30295930

[3] Dasari A Shen C Halperin D Zhao B Zhou S Xu Y. Trends in the Incidence, Prevalence, and Survival Outcomes in Patients With Neuroendocrine Tumors in the United States. JAMA Oncol (2017) 3(10):1335–42. 10.1001/jamaoncol.2017.0589 PMC5824320 28448665

[4] Zhang M Zhao P Shi X Zhao A Zhang L Zhou L. Clinicopathological Features and Prognosis of Gastroenteropancreatic Neuroendocrine Neoplasms in a Chinese Population: A Large, Retrospective Single-Centre Study. BMC Endocr Disord (2017) 17(1):39. 10.1186/s12902-017-0190-6 28705205 PMC5508659

[5] Kulke MH Anthony LB Bushnell DL de Herder WW Goldsmith SJ Klimstra DS. NANETS Treatment Guidelines: Well-Differentiated Neuroendocrine Tumors of the Stomach and Pancreas. Pancreas (2010) 39(6):735–52. 10.1097/MPA.0b013e3181ebb168 PMC3100728 20664472

[6] Delle Fave G Kwekkeboom DJ Van Cutsem E Rindi G Kos-Kudla B Knigge U. Enets Consensus Guidelines for the Management of Patients With Gastroduodenal Neoplasms. Neuroendocrinology (2012) 95(2):74–87. 10.1159/000335595 22262004

[7] Delle Fave G O’Toole D Sundin A Taal B Ferolla P Ramage JK. Enets Consensus Guidelines Update for Gastroduodenal Neuroendocrine Neoplasms. Neuroendocrinology (2016) 103(2):119–24. 10.1159/000443168 26784901

[8] Lipinski M Rydzewska G Foltyn W Andrysiak-Mamos E Baldys-Waligorska A Bednarczuk T. Gastroduodenal Neuroendocrine Neoplasms, Including Gastrinoma - Management Guidelines (Recommended by the Polish Network of Neuroendocrine Tumours). Endokrynol Pol (2017) 68(2):138–53. 10.5603/EP.2017.0016 28540972

[9] NCCN. National Comprehensive Cancer Network. (Nccn) Clinical Practice Guidelines in Oncology. Neuroendocrine and Adrenal Tumors, Version 1. 2019 (2019). Available at: https://www.nccn.org/professionals/physician_gls/default.aspx#neuroendocrine.

[10] Hoffmann KM Furukawa M. Duodenal Neuroendocrine Tumors: Classification, Functional Syndromes, Diagnosis and Medical Treatment. Best Pract Res Clin Gastroenterol (2005) 19(5):675–97. 10.1016/j.bpg.2005.05.009 16253893

[11] Scosyrev E Messing J Noyes K Veazie P Messing E. Surveillance Epidemiology and End Results (SEER) Program and Population-Based Research in Urologic Oncology: An Overview. Urologic Oncology: Seminars and Original Investigations (2012) 30(2):126–32. 10.1016/j.urolonc.2009.11.005 20363162

[12] Lu J Zhao YJ Zhou Y He Q Tian Y Hao H. Modified Staging System for Gastric Neuroendocrine Carcinoma Based on American Joint Committee on Cancer and European Neuroendocrine Tumor Society Systems. Br J Surg (2020) 107(3):248–257. 10.1002/bjs.11408 31971627

[13] Nagtegaal ID Odze RD Klimstra D Paradis V Rugge M Schirmacher P. The 2019 WHO Classification of Tumours of the Digestive System. Histopathology (2020) 76(2):182–8. 10.1111/his.13975 PMC7003895 31433515

[14] Pokala SK Zhang C Chen Z Gamboa AM Cristofaro SL Keilin SA. Lymph Node Metastasis in Early Gastric Adenocarcinoma in the United States of America. Endoscopy (2018) 50(5):479–86. 10.1055/s-0043-122379 29228402

[15] Pourmousavi MK Wang R Kerdsirichairat T Kamal A Akshintala VS Hajiyeva G. Comparable Cancer-Specific Mortality of Patients With Early Gastric Cancer Treated With Endoscopic Therapy vs Surgical Resection. Clin Gastroenterol Hepatol (2020) 18(12):2824–32. 10.1016/j.cgh.2020.04.085 32389885

[16] Han X Cui Y Yang C Sun W Wu J Gao Y. Endoscopic Biopsy in Gastrointestinal Neuroendocrine Neoplasms: A Retrospective Study. PloS One (2014) 9(7):e103210–e103210. 10.1371/journal.pone.0103210 25068592 PMC4113367

[17] Li Q-L Zhang Y-Q Chen W-F Xu M-D Zhong Y-S Ma L-L. Endoscopic Submucosal Dissection for Foregut Neuroendocrine Tumors: An Initial Study. World J Gastroenterol (2012) 18(40):5799–806. 10.3748/wjg.v18.i40.5799 PMC3484351 23155323

[18] Park SM Ham JH Kim B-W Kim JS Kim CW Kim JI. Feasibility of Endoscopic Resection for Sessile Nonampullary Duodenal Tumors: A Multicenter Retrospective Study. Gastroenterol Res Pract (2015) 2015:692492–2. 10.1155/2015/692492 PMC4355118 25810715

[19] Goto A Nishikawa J Hideura E Ogawa R Nagao M Sasaki S. Lymph Node Metastasis can be Determined by Just Tumor Depth and Lymphovascular Invasion in Early Gastric Cancer Patients After Endoscopic Submucosal Dissection. Eur J Gastroenterol Hepatol (2017) 29(12):1346–50. 10.1097/meg.0000000000000987 PMC5690300 29084076

[20] Weatherall T Denbo J Sharpe J Martin M O’Brien T Gupta R. Well-Differentiated, Non-Functional, non-Ampullary Duodenal Neuroendocrine Tumors: Toward Defining Evaluation and Management. World J Surg (2017) 41(3):844–50. 10.1007/s00268-016-3770-0 27743074

[21] Rossi RE Rausa E Cavalcoli F Conte D. Duodenal Neuroendocrine Neoplasms: A Still Poorly Recognized Clinical Entity. Scand J Gastroenterol (2018) 53(7):835–42. 10.1080/00365521.2018.1468479 29726295

[22] Chin JL. Diagnosis and Management of Upper Gastrointestinal Neuroendocrine Tumors. Clin Endos (2017) 50(6):520–9. 10.5946/ce.2017.181 PMC5719910 29207862

[23] Gincul R Ponchon T Napoleon B Scoazec JY Guillaud O Saurin JC. Endoscopic Treatment of Sporadic Small Duodenal and Ampullary Neuroendocrine Tumors. Endoscopy (2016) 48(11):979–86. 10.1055/s-0042-112570 27494453

[24] Draganov PV Wang AY Othman MO. Aga Institute Clinical Practice Update: Endoscopic Submucosal Dissection in the United States. Clin Gastroenterol Hepatol (2019) 17(1):16–25.e11. 10.1016/j.cgh.2018.07.041 30077787

[25] Yazici C. Evolving Role of the Endoscopist in Management of Gastrointestinal Neuroendocrine Tumors. World J Gastroenterol (2017) 23(27):4847–55. 10.3748/wjg.v23.i27.4847 PMC5526755 28785139

[26] Scherubl H Cadiot G Jensen RT Rosch T Stolzel U. Neuroendocrine Tumors of the Stomach (Gastric Carcinoids) are on the Rise: Small Tumors, Small Problems? Endoscopy (2010) 42(8):664–71. 10.1055/s-0030-1255564 20669078

[27] Kwon YH Jeon SW Kim GH Kim JI Chung IK Jee SR. Long-Term Follow Up of Endoscopic Resection for Type 3 Gastric NET. World J Gastroenterol (2013) 19(46):8703–8. 10.3748/wjg.v19.i46.8703 PMC3870517 24379589

[28] Min BH Hong M Lee JH Rhee PL Sohn TS Kim S. Clinicopathological Features and Outcome of Type 3 Gastric Neuroendocrine Tumours. Br J Surg (2018) 105(11):1480–6. 10.1002/bjs.10901 29893418

[29] Noh GY Ku HR Kim YJ Park SC Kim J Han CJ. Clinical Outcomes of Early Gastric Cancer With Lymphovascular Invasion or Positive Vertical Resection Margin After Endoscopic Submucosal Dissection. Surg Endosc (2015) 29(9):2583–9. 10.1007/s00464-014-3973-0 25480609

