# Supplementary figures and images for: Patterns of Lymph Node Metastasis in Patients With T1/T2 Gastroduodenal Neuroendocrine Neoplasms: Implications for Endoscopic Treatment
Source: Front Endocrinol (Lausanne). 2021 May 28;12:658392. doi: 10.3389/fendo.2021.658392 (PMC8194267; doi:10.3389/fendo.2021.658392)

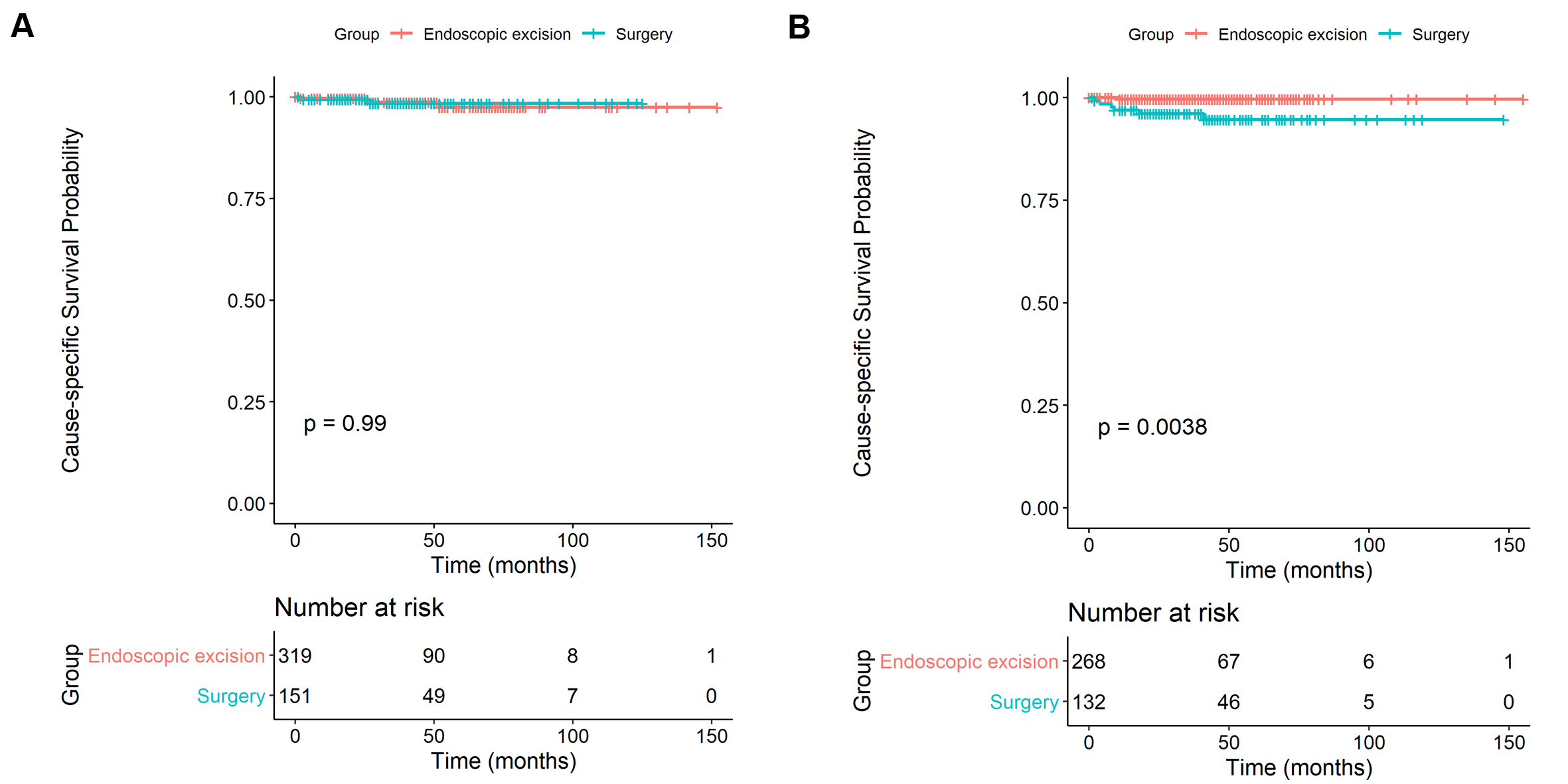

Supplement: Supplementary Figure 1 — Cause-specific survival of nodal-negative g-NETs (A) and d-NETs (B) in the submucosal layer in groups that received endoscopic resection or open surgical resection. g-NET, gastric neuroendocrine tumor; d-NET, duodenal neuroendocrine tumor. [file Image_1.tif]
