# Supplementary material for: Patterns of Lymph Node Metastasis in Patients With T1/T2 Gastroduodenal Neuroendocrine Neoplasms: Implications for Endoscopic Treatment
Source: Front Endocrinol (Lausanne). 2021 May 28;12:658392. doi: 10.3389/fendo.2021.658392 (PMC8194267; doi:10.3389/fendo.2021.658392)
Supplement: Supplementary Table 1A — Cox proportional hazards model assessing factors associated with OS and CSS in T1/T2 g-NETs. [file Table_1.docx]

**Supplementary Table 1A.** Cox proportional hazards model assessing factors associated with OS and CSS in T1/T2 g-NETs.

| **Variable** | **OS** | | |  |  | **CSS** | | | |
| --- | --- | --- | --- | --- | --- | --- | --- | --- | --- |
|  | **Crude HR (95% CI)** | ***P*** | **Adjusted HR (95% CI)** | ***P*** |  | **Crude HR (95% CI)** | ***P*** | **Adjusted HR (95% CI)** | ***P*** |
| Age (years) | 1.00 (0.98-1.02) | 0.88 | 1.00 (0.98-1.02) | 0.97 | | 1.01 (0.98-1.04) | 0.51 | 1.02 (0.99-1.05) | 0.27 |
| Sex (male) | 0.73 (0.45-1.16) | 0.18 | 0.70 (0.43-1.13) | 0.14 | | 0.64 (0.28-1.44) | 0.28 | 0.69 (0.30-1.56) | 0.37 |
| T2 vs T1 stage | 1.38 (0.89-2.13) | 0.15 | 1.48 (0.95-2.31) | 0.08 | | 1.07 (0.51-2.27) | 0.86 | 1.41 (0.65-3.04) | 0.38 |
| Nodal involvement | 2.27 (1.20-4.31) | 0.01 | 2.28 (1.20-4.35) | 0.01 | | 6.05 (2.66-13.8) | < 0.001 | 6.15 (2.67-14.1) | < 0.001 |

Cohort size, n = 706.

**Supplementary Table 1B.** Cox proportional hazards model assessing factors associated with OS and CSS in T1/T2 d-NETs.

| **Variable** | **OS** | | |  |  | **CSS** | | | |
| --- | --- | --- | --- | --- | --- | --- | --- | --- | --- |
|  | **Crude HR (95% CI)** | ***P*** | **Adjusted HR (95% CI)** | ***P*** |  | **Crude HR (95% CI)** | ***P*** | **Adjusted HR (95% CI)** | ***P*** |
| Age (years) | 1.05 (1.03-1.08) | < 0.001 | 1.05 (1.03-1.08) | < 0.001 | | 1.01 (0.97-1.06) | 0.56 | 1.03 (0.98-1.08) | 0.28 |
| Sex (male) | 0.81 (0.49-1.35) | 0.42 | 0.86 (0.52-1.44) | 0.57 | | 0.96 (0.34-2.75) | 0.95 | 1.14 (0.39-3.33) | 0.81 |
| T2 vs T1 stage | 1.06 (0.63-1.79) | 0.83 | 1.06 (0.61-1.85) | 0.83 | | 1.88 (0.66-5.35) | 0.24 | 1.34 (0.43-4.14) | 0.62 |
| Nodal involvement | 1.27 (0.64-2.51) | 0.50 | 1.56 (0.75-3.27) | 0.24 | | 4.00 (1.34-11.9) | 0.01 | 4.25 (1.24-14.5) | 0.02 |

Cohort size, n = 621.

Abbreviations: OS: overall survival; CSS: cause-specific survival; g-NET: gastric neuroendocrine tumor; d-NET: duodenal neuroendocrine tumor; HR: hazard ratio; CI: confidence interval.
