# Supplementary material for: Patterns of Lymph Node Metastasis in Patients With T1/T2 Gastroduodenal Neuroendocrine Neoplasms: Implications for Endoscopic Treatment
Source: Front Endocrinol (Lausanne). 2021 May 28;12:658392. doi: 10.3389/fendo.2021.658392 (PMC8194267; doi:10.3389/fendo.2021.658392)
Supplement: Supplementary Table 2A — Cox proportional hazards model assessing factors associated with OS and CSS in nodal-negative g-NETs confined to the submucosa. [file Table_2.docx]

**Supplementary Table 2A.** Cox proportional hazards model assessing factors associated with OS and CSS in nodal-negative g-NETs confined to the submucosa.

| **Variable** | **OS** | | |  |  | **CSS** | | | |
| --- | --- | --- | --- | --- | --- | --- | --- | --- | --- |
|  | **Crude HR (95% CI)** | ***P*** | **Adjusted HR (95% CI)** | ***P*** |  | **Crude HR (95% CI)** | ***P*** | **Adjusted HR (95% CI)** | ***P*** |
| Age (years) | 1.09 (1.06-1.12) | < 0.001 | 1.09 (1.06-1.12) | < 0.001 | | 1.08 (1.02-1.15) | 0.02 | 1.09 (1.02-1.16) | 0.02 |
| Sex (male) | 1.65 (0.84-3.25) | 0.15 | 1.90 (0.93-3.87) | 0.08 | | 1.04 (0.19-5.68) | 0.96 | 0.99 (0.15-6.51) | 0.99 |
| Tumor size |  |  |  |  | |  |  |  |  |
| ≤ 10 mm | 1 [Reference] |  | 1 [Reference] |  | | 1 [Reference] |  | 1 [Reference] |  |
| 11- 20 mm | 0.87 (0.33-2.28) | 0.78 | 0.81 (0.27-2.46) | 0.71 | | 3.03 (0.51-18.1) | 0.23 | 5.24 (0.60-46.1) | 0.14 |
| > 20 mm | 1.19 (0.36-3.94) | 0.76 | 0.99 (0.29-3.39) | 0.99 | | 3.30 (0.34-31.8) | 0.30 | 3.13 (0.31-31.8) | 0.33 |
| Treatment modalities |  |  |  |  | |  |  |  |  |
| Endoscopic excision | 1 [Reference] |  | 1 [Reference] |  | | 1 [Reference] |  | 1 [Reference] |  |
| Surgery | 0.96 (0.47-1.97) | 0.91 | 1.11 (0.47-2.60) | 0.81 | | 0.99 (0.18-5.39) | 0.99 | 0.55 (0.07-4.59) | 0.58 |

Cohort size, n = 470.

**Supplementary Table 2B.** Cox proportional hazards model assessing factors associated with OS and CSS in nodal-negative d-NETs confined to the submucosa.

| **Variable** | **OS** | | |  |  | **CSS** | | | |
| --- | --- | --- | --- | --- | --- | --- | --- | --- | --- |
|  | **Crude HR (95% CI)** | ***P*** | **Adjusted HR (95% CI)** | ***P*** |  | **Crude HR (95% CI)** | ***P*** | **Adjusted HR (95% CI)** | ***P*** |
| Age (years) | 1.02 (0.99-1.05) | 0.29 | 1.02 (0.99-1.05) | 0.28 | | 0.97 (0.91-1.04) | 0.36 | 0.98 (0.92-1.05) | 0.51 |
| Sex (male) | 0.97 (0.48-1.94) | 0.93 | 0.99 (0.49-1.99) | 0.98 | | 2.43 (0.47-12.5) | 0.29 | 3.03 (0.58-15.7) | 0.19 |
| Tumor size |  |  |  |  | |  |  |  |  |
| ≤ 10 mm | 1 [Reference] |  | 1 [Reference] |  | | 1 [Reference] |  | 1 [Reference] |  |
| >10 mm | 1.03 (0.42-2.51) | 0.95 | 0.98 (0.39-2.43) | 0.96 | | 0.82 (0.10-6.80) | 0.85 | 0.50 (0.06-4.17) | 0.52 |
| Treatment modalities |  |  |  |  | |  |  |  |  |
| Endoscopic excision | 1 [Reference] |  | 1 [Reference] |  | | 1 [Reference] |  | 1 [Reference] |  |
| Surgery | 1.28 (0.63-2.60) | 0.49 | 1.37 (0.66-2.82) | 0.39 | | 11.7 (1.41-97.2) | 0.02 | 13.9 (1.64-117.9) | 0.02 |

Cohort size, n = 400.

Abbreviations: OS: overall survival; CSS: cause-specific survival; g-NET: gastric neuroendocrine tumor; d-NET: duodenal neuroendocrine tumor; HR: hazard ratio; CI: confidence interval.
